# Dual PCR–Sanger Sequencing Assay for Simultaneous *sqle*-Based Identification and Resistance Detection in *Trichophyton* Species

**DOI:** 10.3201/eid3209.260819

**Published:** 2026-09

**Authors:** Nissrine Abou-Chakra, Karin M. Jørgensen, Karen Marie Thyssen Astvad, Maiken Cavling Arendrup

**Affiliations:** Statens Serum Institut, Copenhagen, Denmark (N. Abou-Chakra, K.M. Jørgensen, K.M.T. Astvad, M.C. Arendrup); Rigshospitalet, Copenhagen (M.C. Arendrup); University of Copenhagen, Copenhagen (M.C. Arendrup)

**Keywords:** fungi, sexually transmitted infections, dermatophytosis, terbinafine resistant, sqle target gene, molecular detection

## Abstract

*Trichophyton* fungal infections are common and increasingly complicated by terbinafine resistance. To support accurate molecular identification and resistance profiling, we provide a validated dataset of full-length squalene epoxidase (*sqle*) gene sequences from 493 *Trichophyton* isolates representing 18 species, addressing limited availability and misannotation of *sqle* sequences in public databases, particularly within the *T. mentagrophytes* complex. On the basis of that dataset, we designed 2 Sanger sequencing assays for simultaneously identifying species and detecting resistance-associated mutations. One targets the full-length gene, whereas the other amplifies a short region capturing all key resistance hotspots and species-specific single-nucleotide polymorphisms. The partial assay was evaluated on 66 clinical specimens and enabled clinically relevant species identification without internal transcribed spacer sequencing, supporting culture-independent detection. Of note, this approach differentiates *T. indotineae* from closely related species, overcoming a key diagnostic limitation and supporting clinical decision-making, surveillance, and management of resistant infections, despite limited resolution within certain species complexes.

*Trichophyton* species fungi cause infections of the skin, nails, and hair and are estimated to affect 20%–25% of persons during their lifetimes (*1*). Clinical manifestations include pruritus, erythema, scaling, alopecia, and inflammation (*2*). Although infections are often considered minor, chronic or extensive dermatophytosis can cause considerable illness, involving prolonged treatment, increased healthcare use, and impaired quality of life (*3*). The recent rising prevalence of terbinafine-resistant *Trichophyton* strains is associated with therapeutic failure, underscoring the need for rapid and reliable diagnostics to detect resistance, guide therapy, and thereby limit transmission (*4*–*6*).

Traditional culture-based diagnostics are slow and exhibit suboptimal sensitivity, especially in onychomycosis or after antifungal therapy (*7*). Molecular assays targeting the internal transcribed spacer (ITS) region of ribosomal DNA are widely adopted because of its high copy number and potential for improved identification (*8*). However, limited sequence variation in this region hinders reliable discrimination among closely related *Trichophyton* species, particularly within the *T. mentagrophytes* complex (*9*,*10*). Of note, *T. indotineae*, an emerging and often terbinafine-resistant species within this complex, might be misidentified by certain ITS-based diagnostic kits and commercial platforms such as DermaGenius 2.0 (PathoNostics, https://www.pathonostics.com) (*11*–*16*). Those challenges are further compounded by recent taxonomic revisions and inconsistencies in available reference databases (*9*,*12*).

The *sqle* gene (alternatively, *erg1*) encodes squalene epoxidase (SQLE), the molecular target of terbinafine. Mutations at conserved hotspot positions (e.g., L393, F397, F415, and H440) reduce drug binding while preserving enzymatic function, thereby conferring terbinafine resistance (*17*). Recent studies from India, Europe, and North America document the global increase of resistant SQLE variants, particularly in *T. indotineae* and *T. rubrum* (*18*–*21*). However, the absence of robust, curated, and taxonomically validated *sqle* reference sequences limits the diagnostic and surveillance utility of this gene.

In this study, we generated a curated, taxonomically validated dataset of full-length *sqle* sequences, supported by reliable ITS-based identification, to address limitations in existing databases. We also present a sequencing-based workflow enabling simultaneous species identification and detection of terbinafine resistance-conferring *sqle* mutations in *Trichophyton* directly from clinical specimens or isolates.

## Materials and Methods

### Strains and Clinical Samples

We included a total of 493 clinical *Trichophyton* isolates (Table 1; Appendix Table 1), collected during 2022–2025 and representing 18 species. We confirmed species identification by ITS sequencing, showing 100% identity to reference sequences (*4*,*16*,*22*) (Appendix Table 2). *T. schoenleinii* was unavailable in culture but was included on the basis of its *sqle* sequence, which we retrieved from GenBank (accession no. GCA_018357685.2). We used the *sqle* sequence for *Microsporum canis* as an outgroup for phylogenetic rooting, following the procedures described in the CLC Genomics Workbench manual 24.0.4 (QIAGEN,https://www.qiagen.com). In addition, we included 66 clinical specimens (skin scrapings, nail clippings, scalp, and hair) that previously tested positive for *Trichophyton* spp. using an in-house ITS-based PCR (*23*) that does not distinguish among *T. mentagrophytes*, *T. interdigitale*, and *T. indotineae*.

**Table 1:** MICs determined using the EUCAST E.Def 11.0 reference method for dermatophytes in study of dual PCR–Sanger sequencing assay for simultaneous *sqle*-based identification and resistance detection in *Trichophyton* species†

Species	SQLE amino acid alteration (intrinsic SNPs)	Terbinafine MIC, mg/L		No. isolates
		Modal MIC‡	Range	
*T. indotineae*, n = 105	WT	ND	0.03–0.06	3
	L393F	ND	>4	1
	L393S	ND	0.5–1	3
	F397L	>4	2–>4	63
	F397L/Y414H	ND	>4	1
	F397L/A448T	ND	4–>4	6
	Q408L	2	1–4	20
	F415C	ND	1	1
	F415L	ND	0.5	1
	A448T	ND	0.03–0.125	6
*T. interdigitale*, n = 26	WT	0.008/0.016	<0.004-0.06	21
	L393F	ND	>4	1
	L393S	ND	0.25–1	3
	F415L	ND	0.25	1
*T. mentagrophytes* ITS genotype DK-I, n = 1§	WT (SNPs: N276¶, F419 and T454)	ND	0.06	1
*T. mentagrophytes* ITS genotype DK-II, n = 4§	WT (SNPs: N276¶, F419 and P454)	ND	0.008–0.06	4
*T. mentagrophytes* ITS genotype III, n = 2§	WT (SNPs: N276¶, F419 and T454)	ND	0.016–0.03	2
*T. mentagrophytes* ITS genotype III*, n = 9§	WT (SNPs: N276¶, F419 and T454)	ND	0.008–0.03	9
*T. mentagrophytes* ITS genotype IV, n = 1§	WT (SNPs: K276¶, L419 and T454)	ND	0.03	1
*T. mentagrophytes* ITS genotype VII, n = 2§	WT (SNPs: N276¶, F419 and T454)	ND	0.016–0.03	2
*T. rubrum*, n = 285	WT	0.016	<0.004–0.06	143
	Y110C¶	ND	0.008	1
	I121M¶	ND	0.03	1
	I121M¶/F415S	ND	0.5	1
	L335F¶/F415S	ND	2	1
	L393del	ND	4	2
	L393F	ND	>4	7
	L393F/Y394del	ND	>4	2
	L393S	0.5	0.25–2	15
	S395_I396insS	ND	0.5	3
	F397I	1	0.25–1	11
	F397L	4	1->4	78
	Y414H	ND	0.03–0.06	2
	F415L	ND	0.125	1
	F415S	ND	0.125–1	3
	H440Y	ND	0.016–0.125	9
	A448T	ND	0.03	1
	A448V	ND	0.004	1
	L451P	ND	0.125	1
	C476Y	ND	0.016–0.03	2
*T. soudanense*, n = 2§	WT	ND	<0.004–0.008	2
*T. tonsurans*, n = 16§	WT	0.016	0.008–0.06	16
*T. violaceum*, n = 24§	WT	0.03	0.004–0.125	24
*T. equinum,* n = 1§	WT	ND	0.03	1
*T. simii*, n = 1§	WT	ND	<0.004	1
*T. erinacei,* n = 2§#	WT	ND	Not tested	2
*T. concentricum,* n = 1§#	WT	ND	Not tested	1
*T. europaeum*, n = 1§	WT	ND	0.03	1
*T. benhamiae var. benhamiae/var. luteum*, n = 10§	WT	ND	0.004–0.06	10

†EUCAST tentative epidemiological cutoff values for terbinafine (0.125 mg/L for *T. indotineae* and 0.03 mg/L for *T. rubrum*) were used to classify isolates as wild-type or non–wild-type. Underlined SQLE alterations are associated with terbinafine MICs above the corresponding tentative epidemiological cutoff values. An expanded version of this table appears in the Appendix. DK-I, unassigned I; DK-II, unassigned II; EUCAST, European Committee on Antimicrobial Susceptibility Testing; ITS, internal transcribed spacer; ND, not determined; SNP, single-nucleotide polymorphism; SQLE, squalene epoxidase; WT, wild-type. ‡Modal MIC is reported only for distributions comprising >10 isolates. Bimodal distributions are reported as both modal MICs. §Indicates that all isolates of the indicated species were wild-type. DK-I and DK-II denote novel ITS genotypes identified in this study. ¶Indicates that the codon is not covered by sequencing of the short *sqle* fragment (spanning amino acids H376–V483). #EUCAST susceptibility testing was not performed.

### DNA Extraction

For fungal isolates, we suspended colonies in 400 µL of NUCLISENS Lysis Buffer (bioMérieux, https://www.biomerieux.com), of which we used 200 µL for DNA extraction on the automated eMAG system (bioMérieux) according to protocol B, which includes automated nucleic acid purification to reduce the presence of potential PCR inhibitors. We eluted DNA in 100 µL of NUCLISENS Extraction Buffer 3 (bioMérieux). We incubated clinical samples overnight at 56 °C in 1,000 µL NUCLISENS Lysis Buffer with 50 µL proteinase K (QIAGEN). Subsequently, we added 100 µL of 1 M dithiothreitol (DTT) (Sigma-Aldrich) and incubated samples for 30 minutes before centrifugation at 22,000 × *g* for 5 minutes. We then subjected 1,000 µL of clarified supernatant to extraction on the eMAG system according to protocol B and eluted it in 50 µL of NUCLISENS Extraction Buffer 3. Each run included a negative extraction control. We stored extracted DNA at −20 °C until further analysis.

### Antifungal Susceptibility Testing

We determined MICs in RPMI 1640 medium according to the EUCAST E.Def 11.0 reference method (*24*). We prepared terbinafine (final concentration after inoculation 0.004–4 mg/L) from frozen stock solutions. We adopted EUCAST tentative epidemiologic cutoff values (TECOFFs) of terbinafine as 0.125 mg/L for *T. indotineae* and 0.03 mg/L for *T. rubrum* for classification as wild-type or non–wild-type (*25*).

### Amplification and Sequencing of *sqle*

Two PCR strategies enabled species identification and detection of resistance-associated mutations: full-length *sqle* amplification and sequencing for cultured isolates and partial *sqle* amplification for clinical specimens. All primers were *Trichophyton*-specific. Each run included a positive fungal DNA control and a no-template control. PCR product purification and sequencing were performed by Macrogen Europe BV (https://www.macrogen-europe.com).

#### Full-Length *sqle *

We performed amplification using primers TRI-SE-F (5′-GGTGACAGCGACAAGTGCC-3′) and TRI-SE-R (5′-AGTCCAACCRACCCCAGTCA-3′; R = A/G). PCR reactions (25 µL) contained 2 µL genomic DNA, 0.4 µM of each primer, 12.5 µL Extract-N-Amp PCR ReadyMix (Sigma-Aldrich, https://www.sigmaaldrich.com), and 8.5 µL UltraPure DNase/RNase-free water (Thermo Fisher Scientific, https://www.thermofisher.com). Cycling conditions were 95°C for 30 seconds, followed by 35 cycles of 95°C for 30 seconds, 57°C for 45 seconds, and 72°C for 90 seconds. We performed bidirectional sequencing using the amplification primers and internal primers TRI-SE-S1 (5′-GGAATATCTCCCCATACAACCAG-3′) and TRI-SE-S2 (5′-AACGCAGCTTCAAAYGAGGG-3′; Y = C/T).

#### Partial *sqle*

We amplified a 388-bp fragment spanning positions 1125–1512 of the full-length *sqle* sequence and encompassing clinically relevant terbinafine resistance–associated mutations, as well as species-specific single-nucleotide polymorphisms (SNPs) for species-level identification, using primers F0 (5′-CCACTGGCAACGGAAGTC-3′) and R0 (5′-ATACGAAAGGAAGGATGACCC-3′). PCR reactions (25 µL) contained 5 µL template DNA, 0.4 µM of each primer, 12.5 µL Extract-N-Amp PCR ReadyMix (Sigma-Aldrich), and 5.5 µL UltraPure DNase/RNase-free water. Cycling conditions were 95°C for 30 seconds, followed by 35 cycles of 95°C for 30 seconds, 58°C for 45 seconds, and 72°C for 90 seconds. We performed sequencing using the amplification primers.

#### Sequence Analysis and Phylogenetics 

We quality-checked, trimmed, and assembled raw reads; we aligned full-length sequences using CLC Main Workbench (QIAGEN). Species and genotype assignments were based on sequence alignment and comparison of informative species- and genotype-specific SNP profiles within the analyzed region. We identified terbinafine resistance–associated mutations by evaluation of nucleotide substitutions at previously described SQLE resistance hotspots. We calculated pairwise SNP differences and reconstructed phylogenetic relationships using the neighbor-joining algorithm with the Kimura 2-parameter (K2P/K80) model. We evaluated node support by 1,000 bootstrap replicates.

## Results

### Complete Intraspecies Conservation in Wild-Type Isolates

We obtained full-length *sqle* sequences for 493 terbinafine wild-type and non–wild-type isolates (Table 1; Appendix Table 1) representing phylogenetically and epidemiologically relevant taxa and aligned them within each taxon to assess intraspecific conservation. We identified high-frequency wild-type *sqle* alleles in 244 terbinafine-susceptible isolates, including *T. rubrum* (143/285), *T. interdigitale* (21/26), and *T. indotineae* (3/105), all showing complete sequence conservation within each species. The same pattern was also observed for all ITS genotypes of *T. mentagrophytes* (n = 19), *T. violaceum* (n = 24), *T. benhamiae* (n = 10), *T. tonsurans* (n = 16), and other less common *Trichophyton* species.

Only *sqle* sequences from isolates with wild-type MICs and no SQLE amino acid alterations were included in the curated reference dataset. A strict wild-type consensus was defined for each taxon. Those sequences have been deposited in GenBank (accession nos. PZ614143–PZ614159 and PX205352) and, together with the *sqle* sequence of *T. schoenleinii*, constitute the final reference dataset (Appendix Table 2).

We analyzed the spectrum of SQLE amino acid alterations and corresponding MICs in the remaining isolates. We observed low-frequency variants (A448T, A448V, Y110C, C476Y, and I121M) sporadically (n = 12); those variants were confined to *T. rubrum* and *T. indotineae* isolates with MICs within the species-specific wild-type range. The Y414H substitution was detected in 2 *T. rubrum* isolates with MICs around the TECOFF. Those variants were not considered part of the predominant wild-type allele population. The remaining 235 isolates harbored missense mutations affecting hotspot amino acid residues within the catalytic domain of SQLE and were associated with elevated terbinafine MICs (Table 1; Appendix Table 1).

### Species-Level Discrimination of *Trichophyton* Using Full-Length *sqle* Sequencing

**Figure 1:**
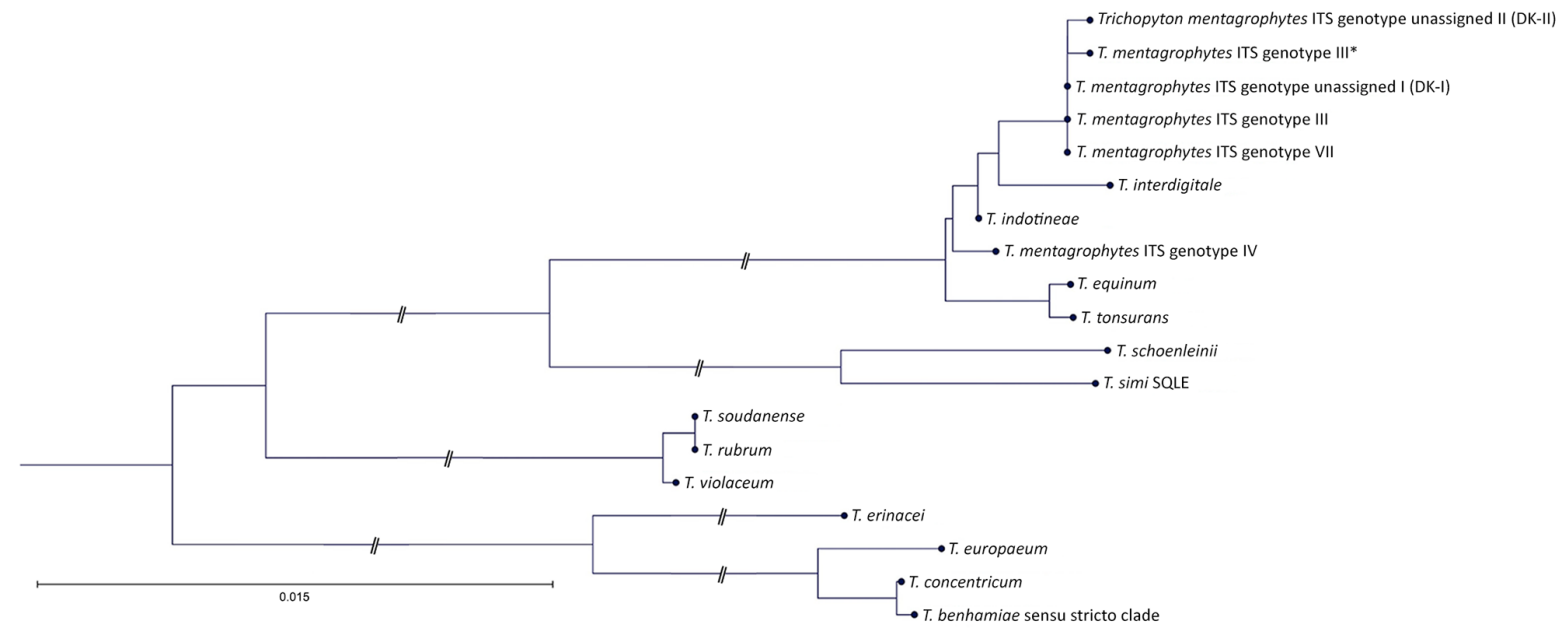
Phylogenetic tree of *Trichophyton* species inferred from sequence variation in the full-length squalene epoxidase (*sqle*) gene in study of dual PCR–Sanger sequencing assay for simultaneous *sqle*-based identification and resistance detection in *Trichophyton* species. Within the *T. mentagrophytes* species complex, isolates identified as *T. mentagrophytes* represented ITS genotypes III, III*, IV, and VII. The remaining *T. mentagrophytes* isolates formed 2 consensus sequences that did not show 100% identity to any sequence in GenBank and were designated DK-I and DK-II. Scale bar indicates number of substitutions per site.

Pairwise comparisons of full-length *sqle* consensus sequences revealed substantial interspecies SNP variation across *Trichophyton*, enabling discrimination at multiple taxonomic levels (Appendix Table 3, Figure 1). Polymorphisms were distributed throughout the gene, with species-specific and genotype-specific patterns capturing both interspecies and intracomplex diversity. SNP differences ranged from complete sequence identity (0 SNPs) among closely related taxa to 92 SNPs within *Trichophyton* and 341–351 SNPs relative to the outgroup *M. canis*. Phylogenetic analyses showed clear separation between species and major clades (Figure 1).

The *T. rubrum* complex was distinct from the *T. mentagrophytes* complex and other species (≈65–75 SNPs). Of note, *T. indotineae*, within the *T. mentagrophytes* complex, differed from the *T. rubrum* complex by 65–66 SNPs distributed across the full-length *sqle* gene, including 6 resulting in amino acid substitutions. Within the *T. rubrum* complex, divergence was minimal: *T. rubrum* and *T. soudanense* shared identical sequences, whereas *T. violaceum* differed by 2 SNPs (positions 186 and 522), the latter resulting in a nonsynonymous substitution. *T. tonsurans* and *T. equinum* formed a closely related pair, differing by only 2 SNPs (positions 117 and 1256), but diverged from *T. indotineae* by 7 SNPs*,* from the *T. mentagrophytes* complex by 8–12 SNPs, and from *T. interdigitale* by 13 SNPs.

More distantly related species showed >60 SNP differences relative to both the *T. mentagrophytes* and *T. rubrum* complexes and were individually clearly separable. *T. simii* and *T. schoenleinii* differed by 24 SNPs, 4 of which were nonsynonymous. Within the *T. benhamiae* complex, sequence diversity was notable. *T. concentricum*, the only strictly anthropophilic species within the complex (*26*), differed from *T. benhamiae* by a single SNP, whereas *T. europaeum* differed by 10 SNPs and *T. erinacei* differed by 42 SNPs.

Within closely related taxa of the *T. mentagrophytes* complex, SNP differences were lower but remained structured. *T. interdigitale* showed the greatest divergence, differing from *T. indotineae* by 6 synonymous SNPs (positions 169, 642, 882, 1251, 1457, and 1460) and from other genotypes by 8 to 9 SNPs, comprising both synonymous and nonsynonymous changes.

Similarly, *T. indotineae* differed from other genotypes within the *T. mentagrophytes* complex by 3–5 SNPs, reflecting a structured pattern of variation. It differed from unassigned lineage II and genotype III* by 5 SNPs (3 synonymous: 717, 996, 1251; 2 nonsynonymous: 828, 1317), whereas divergence from unassigned lineage I and genotypes III and VII was limited to 4 SNPs at the same loci; position 717 was conserved across those groups. The closest relationship was observed with genotype IV, from which *T. indotineae* differed by only 3 synonymous SNPs (positions 246, 813, 1391).

Within the *T. mentagrophytes* complex, unassigned lineage I and genotypes III and VII shared identical *sqle* sequences. Those showed limited variation relative to unassigned lineage II, genotype III*, and genotype IV. Genotype IV was the most divergent, differing by 7–8 SNPs from the other genotypes. In contrast, genotype III* and unassigned lineage II each differed from the identical group by a single synonymous SNP at position 717 for genotype III* and position 1422 for unassigned lineage II. Although the number of SNP differences among closely related members of the *T. mentagrophytes* complex was limited, distinct SNP profiles enabled discrimination of *T. indotineae*, *T. interdigitale*, and the investigated lineages/genotypes (Appendix Table 4).

### Lineage-Specific Variation Within the *T. mentagrophytes* Complex

**Figure 2:**
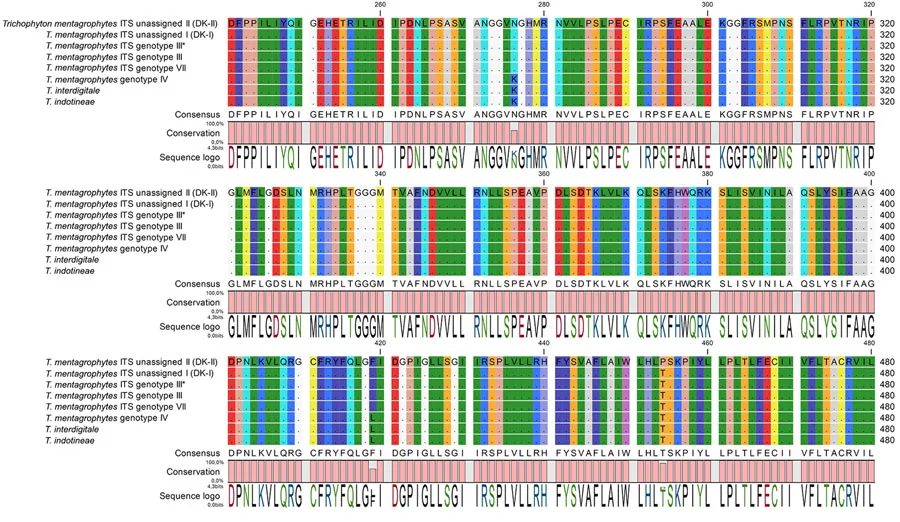
Amino acid polymorphisms in squalene epoxidase within the *T. mentagrophytes* complex in the study of dual PCR-Sanger sequencing assay for simultaneous *sqle*-based identification and resistance detection in *Trichophyton* species. Alignment of SQLE amino acid sequences showing lineage-specific patterns: N276/F419 (ITS genotypes III/III*/VII and unassigned genotypes [DK-I–II]), P454 unique to DK-II, and K276/L419 in ITS genotype IV (shared with *T. interdigitale* and *T. indotineae*).

Comparative analysis of full-length 489-aa sequences within the *T. mentagrophytes* complex revealed distinct lineage-associated patterns without effects on the susceptibility (MICs 0.008–0.06 mg/L) (Figure 2). A group of isolates consistently harbored N276 and F419, including 13 isolates assigned to ITS genotypes III (n = 2), III* (n = 9), and VII (n = 2), and 5 unassigned isolates (unassigned lineage I, n = 1; unassigned lineage II, n = 4). In addition to the characteristic N276 and F419 residues, all unassigned lineage II isolates harbored a unique alteration at position P454, in contrast with an alteration at position T454 in all other genotypes and species across the genus. In contrast, the *T. mentagrophytes* ITS genotype IV isolate differed from other genotypes in displaying K276 and L419, a pattern shared with *T. interdigitale* and *T. indotineae*.

### Species Identification Using 388-bp *sqle* Fragment Sequencing

**Figure 3:**
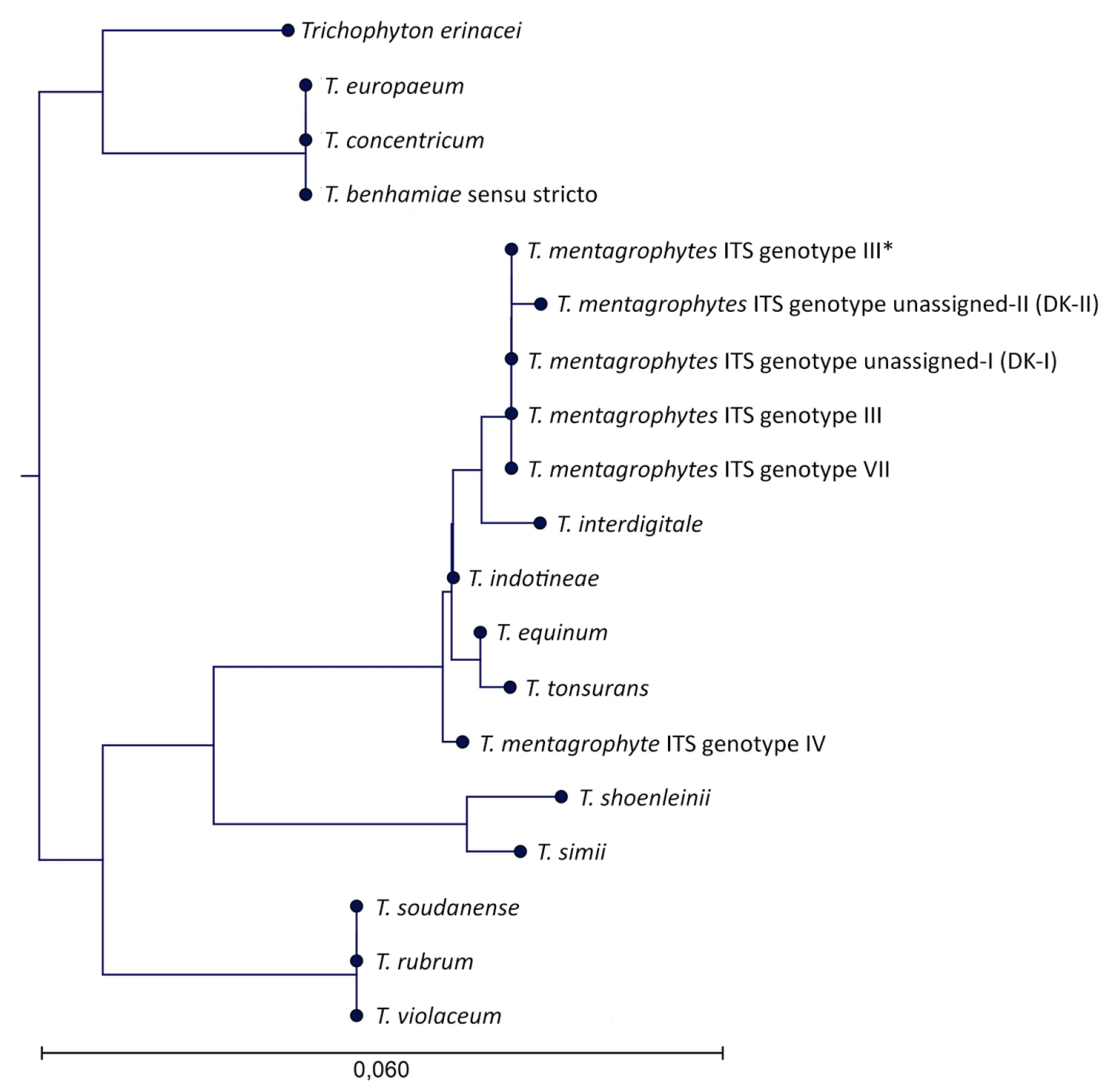
Phylogenetic tree of *Trichophyton* species inferred from sequence differences in the partial 388-bp squalene epoxidase (*sqle*) gene fragment in study of dual PCR–Sanger sequencing assay for simultaneous *sqle*-based identification and resistance detection in *Trichophyton* species. Within the *T. mentagrophytes* species complex, isolates identified as *T. mentagrophytes* represented ITS genotypes III, III*, IV, and VII. The remaining *T. mentagrophytes* isolates formed 2 consensus sequences that did not show 100% identity to any sequence in GenBank and were designated DK-I and DK-II. Scale bar indicates number of substitutions per site.

A second primer set was designed to amplify a 388 bp fragment of the *sqle* gene (≈1531–1534 bp from start to stop codon, depending on species) spanning amino acid residues H376 to V483 within the catalytic domain and including known terbinafine-resistance hotspots (*27*). Despite its short length, this segment exhibited sufficient interspecies SNP variation to enable discrimination of most *Trichophyton* species and to support resistance detection (Appendix Table 5, Figure 2). Phylogenetic reconstruction supported its discriminatory capacity, yielding well-supported clades corresponding to major species complexes (Figure 3). The *T. mentagrophytes* ITS genotypes were clearly delineated from *T. indotineae* and *T. interdigitale*, which were also resolved from each other. The *T. rubrum* complex (*T. rubrum*, *T. violaceum*, and *T. soudanense*) formed a coherent cluster, although species-level resolution was not achieved. Within the *T. benhamiae* clade, *T. erinacei* was clearly differentiated (14 SNPs), whereas *T. europaeum*, *T. benhamiae*, and *T. concentricum* clustered together. Finally, *T. schoenleinii* and *T. simii* formed an independent lineage.

### Clinical Validation of the Partial *sqle* PCR

We applied the partial *sqle* PCR directly to DNA from 66 clinical specimens (skin, hair, and nails) previously positive for *Trichophyton* spp. using an in-house ITS-based PCR unable to distinguish among *T. mentagrophytes*, *T. interdigitale* and *T. indotineae*. All samples produced amplicons of the expected size, enabling sequencing-based species identification as well as detection of resistance-associated mutations (Table 2).

**Table 2:** Species identification and *sqle* alterations detected by direct PCR and sequencing of a partial 388-bp *sqle* gene fragment from clinical specimens positive for *Trichophyton* spp. in study of dual PCR–Sanger sequencing assay for simultaneous *sqle*-based identification and resistance detection in *Trichophyton* species*

Initial ITS-based identification	Specimen types	No. clinical samples	Final species identification (*sqle* sequencing)	SQLE variant(s)
*T. rubrum*	Nail, skin scraping	24	*T. rubrum complex*†	Wild-type, n = 19
				L393S, n = 1
				F397L, n = 2
				F415S, n = 1
				L427M, n = 1
*T. mentagrophytes* complex‡	Nail, skin scraping	28	*T. interdigitale* (n = 16)	Wild-type, n = 16
			*T. mentagrophytes* (n = 1)	Wild-type, n = 1
			*T. indotineae* (n = 11)	Q408L, n = 4
				F397L, n = 3
				A448T, n = 2
				F397L/A448T, n = 1
				Wild-type, n = 1
*T. tonsurans*/*T. equinum*	Hair, skin scraping	4	*T. tonsurans*	Wild-type, n = 4
*T. violaceum*	Hair, scalp	2	*T. rubrum *complex†	Wild-type, n = 2
*Trichophyton* spp.‡	Scalp	4	*T. rubrum *complex†	Wild-type, n = 4
*T. benhamiae complex*	Hair, skin scraping	4	*T. benhamiae *sensu stricto clade	Wild-type, n = 4

*Initial species identification was based on ITS analysis, with final identification resolved by *sqle* sequencing. Detected *sqle* variants include both wild-type and amino acid substitutions within the terbinafine resistance hotspot region. ITS, internal transcribed spacer; SQLE, squalene epoxidase. †Indicates assignment to species complexes where species-level resolution was not achieved. ‡Further identification not possible.

The 28 samples previously identified as *T. mentagrophytes* complex were unambiguously resolved by *sqle* sequencing as *T. interdigitale* (n = 16), *T. indotineae* (n = 11), and *T. mentagrophytes* (n = 1). All *T. interdigitale* samples were wild-type, whereas 10 of 11 *T. indotineae* samples harbored >1 nonsynonymous alterations, including known terbinafine resistance–associated substitutions: Q408L (n = 4), F397L (n = 3), and F397L/A448T (n = 1). In addition, 24 samples from the *T. rubrum* complex were confirmed, of which 19 were wild-type and 5 harbored nonsynonymous substitutions (L393S, n = 1; F397L, n = 2; F415S, n = 1; L427M, n = 1).

We confirmed 4 scalp and skin samples initially identified as *T. tonsurans/T. equinum* as *T. tonsurans*, all with wild-type *sqle* sequences. Two hair and scalp specimens previously identified as *T. violaceum* and 4 additional scalp specimens previously identified only at the genus level were assigned to the *T. rubrum* complex, all without resistance-associated *sqle* mutations. Four hair and skin scraping specimens initially assigned to the *T. benhamiae* complex were resolved as *T. benhamiae* sensu stricto, all with wild-type sequences and distinct from *T. erinacei*.

## Discussion

Early and accurate identification and susceptibility classification of *Trichophyton* isolates are essential for clinical management, source tracing, and epidemiologic surveillance, particularly in the context of terbinafine resistance, highly transmissible anthropophilic lineages, and increased person-to-person transmission, including sexual transmission (*28*–*33*). Morphological differentiation remains challenging because of phenotypic variability and overlap between species (*34*–*36*). Historically, species within the *T. mentagrophytes* complex were grouped broadly, contributing to taxonomic ambiguity. Although molecular approaches improved resolution, taxonomic confusion remained. For example, isolates within the *T. mentagrophytes/interdigitale* complex were often assigned to *T. interdigitale* (*36*), and species from the *T. benhamiae* complex, including *T. erinacei*, were misclassified at *T. mentagrophytes* complex (*26*). The 2017 taxonomy revision by de Hoog et al. (*34*) provided a more structured framework, yet genetic resolution remains challenging. ITS sequencing often lacks discriminatory power within closely related taxa, including the *T. mentagrophytes* and *T. rubrum* complexes, where limited variation does not reflect clinical or epidemiologic diversity (*37*,*38*). That shortfall is particularly relevant for *T. indotineae* (formerly *T. mentagrophytes* type VIII), now recognized as a distinct species of clinical importance (*9*,*36*,*37*), and for type VII, an emerging anthropophilic genotype associated with genital lesions among men who have sex with men (*30*,*33*).

BLAST-based identification is further limited by inconsistent database annotation, lack of standardized genotype classification, and ongoing taxonomic revisions. Consequently, identical ITS sequences might yield multiple high-scoring (even 100%) matches across closely related species, requiring manual curation and preventing reliable species-level identification. Those issues, combined with outdated database entries and limited ITS variability, reduce the utility of ITS-based diagnostics and highlight the need for curated reference databases and complementary genetic markers.

Alternative genetic targets, including translation elongation factor 1-α, β-tubulin, calmodulin, 28S rDNA and mating-type genes, offer improved resolution (*39*–*42*). However, their routine use is limited by less universal primers, the need for species-specific or optimised PCR protocols, limited reference data, and the increased complexity and cost of multilocus sequencing, limiting their applicability in routine diagnostics.

Against this background, we investigated *sqle* as a combined marker for *Trichophyton* identification and terbinafine-resistance detection. Successful amplification and sequencing across all strains generated a robust reference dataset and demonstrated broad primer applicability. Complete intraspecies conservation supports strong functional constraint, whereas interspecies variation enables discrimination of closely related taxa. Phylogenetically, the *T. rubrum* and *T. benhamiae* complexes were clearly distinct from each other and from the *T. mentagrophytes* complex.

Of note, the full-length *sqle* gene enables reliable discrimination of *T. indotineae* and *T. interdigitale* from other members of the *T. mentagrophytes* complex, including genotype IV. That distinction is clinically critical, because both are anthropophilic rather than zoophilic, and *T. indotineae* is frequently terbinafine-resistant and associated with more persistent and outbreak-prone infections. The remaining isolates within *T. mentagrophytes* complex belonged to established genotypes (III, III*, IV, and VII), which are largely associated with distributions in Europe (*43*). Genotype IV showed greater divergence (7–9 SNPs) but remained closest to *T. indotineae* (3 SNPs). Five isolates formed 2 ITS consensus sequences **(**DK-I and DK-II**)** that could not be assigned to any currently reported ITS genotypes; the closest database matches showed only 99.5%–99.6% sequence identity. In contrast, 3 ITS genotypes (DK-I, III, and VII) shared identical *sqle* gene sequences and differed minimally from others, indicating that additional markers such as ITS are required if fine-scale resolution within this complex is required. Additional *sqle* diversity might exist in unrepresented genotypes. The genetic findings were further reflected at the amino acid level: genotype IV shared K276 and L419 with *T. indotineae* and *T. interdigitale*, whereas other genotypes consistently carried N276 and F419. Reports of N276 alone might therefore reflect incomplete *sqle* reference sequences lacking the region encompassing position 419 (*44*). The DK-II isolates harbored a unique P454 substitution (threonine to proline) not observed in other *Trichophyton* species, suggesting additional lineage-level variation, although confirmation requires larger datasets. Of note, lineage-specific polymorphisms such as N276 and F419 have often been reported as the K276N/L419F substitution because of inappropriate *sqle* reference sequences. On the basis of our findings, those variants should be considered wild-type and intrinsic to certain *T. mentagrophytes* genotypes; reported MIC variation more likely reflects methodological differences or genotype-specific susceptibility rather than acquired resistance (*18*,*45*,*46*).

Within the *T. rubrum* complex, *T. rubrum* and *T. violaceum* could be distinguished, whereas *T. soudanense* shared identical *sqle* sequences with *T. rubrum*, indicating that *sqle* alone is insufficient for discrimination and requires complementary ITS sequencing. That limitation also applies to ITS, because species within the *T. rubrum* complex, including *T. violaceum* and *T. soudanense*, also show minimal genetic variation despite being epidemiologically and phenotypically distinct. In contrast, the *T. benhamiae* complex showed greater genetic heterogeneity with *sqle*, providing improved resolution compared with ITS, particularly among *T. benhamiae*, *T. europaeum*, and *T. erinacei*.

Our analysis confirmed that terbinafine non–wild-type *Trichophyton* isolates harbor nonsynonymous *sqle* mutations within the catalytic domain, clustering at residues critical for terbinafine binding (*27*). Resistance in absence of *sqle* mutations appeared rare; only a single *T. rubrum* isolate had an MIC marginally above the TECOFF, which might reflect technical variation associated with MIC testing. All resistant isolates contained >1 alteration within the L393–L451 region.

Targeted amplification of a short *sqle* fragment offers a practical and clinically applicable alternative to full-length sequencing. Despite its reduced size, the 388-bp region retained sufficient phylogenetic information to delineate major *Trichophyton* species and complexes, while capturing resistance-conferring mutations. The shorter amplicon improves PCR efficiency and sensitivity and can be applied directly to clinical specimens, enabling culture-independent detection of both species and resistance. Its ability to distinguish clinically relevant taxa—such as *T. indotineae*, *T. interdigitale*, and the remaining *T. mentagrophytes* genotypes as a single group, as well as the *T. rubrum* and *T. benhamiae* complexes as distinct entities—supports its diagnostic utility, because resolution at this complex level is often sufficient for clinical decision-making. However, discrimination within certain complexes remains limited. Most *T. mentagrophytes* genotypes (DK-I, III, III*, and VII) cluster with identical *sqle* sequences, whereas DK-II was distinguished by 1 SNP and genotype IV was distinguished by 3 SNPs. Similarly, *T. rubrum* and *T. violaceum* (within the *T. rubrum* complex), and *T. benhamiae* sensu stricto, *T. europaeum*, and *T. concentricum* (within the *T. benhamiae* complex) were not fully resolved within their respective clusters.

The first limitation of this study is that although the assay performed well under routine diagnostic conditions, we did not formally evaluate its analytical sensitivity (limit of detection) and specificity, which should be addressed in future validation studies. Moreover, antifungal susceptibility testing was performed only once per isolate. Consequently, technical variation could explain the elevated MIC observed for terbinafine against 1 *T. rubrum* isolate despite its wild-type *sqle* genotype.

In conclusion, *sqle* sequencing enables simultaneous species identification and resistance detection of *Trichophyton* species, supported by a curated reference dataset for reliable interpretation. A short-amplicon approach enables application to clinical specimens and provides information on resistance-associated mutations and clinically relevant species discrimination, although with limited resolution within some complexes. This approach has the potential to enable early optimization of antifungal therapy even in culture-negative cases.

Appendix Additional information about dual PCR–Sanger sequencing assay for simultaneous *sqle*-based identification and resistance detection in *Trichophyton* species.
