# Supplementary material for: Dual PCR–Sanger Sequencing Assay for Simultaneous sqle-Based Identification and Resistance Detection in Trichophyton Species
Source: Emerg Infect Dis. 2026 Sep;32(9):1441–51. doi: 10.3201/eid3209.260819 (PMC13555740; doi:10.3201/eid3209.260819)
Supplement: Supplementary file 1 [file 26-0819-Techapp-s1.pdf]

Article DOI: https://doi.org/10.3201/eid3209.260819

*EID cannot ensure accessibility for supplementary materials supplied by authors. Readers who have difficulty accessing supplementary content should contact the authors for assistance.*

# Dual PCR–Sanger Sequencing Assay for Simultaneous *sqle*-Based Identification and Resistance Detection in *Trichophyton* Species

## Appendix

**Appendix Table 1.** MICs were determined using the EUCAST E.Def 11.0 reference method for dermatophytes. EUCAST tentative epidemiologic cutoff values (TECOFFs) for terbinafine (0.125 mg/L for *T. indotineae* and 0.03 mg/L for *T. rubrum*; EUCAST, 2025) were used to classify isolates as wild-type or non-wild-type. Underlined SQLE alterations correspond to elevated terbinafine MICs . An [a] indicates that the codon is not covered by the short *sqle* fragment sequencing (spanning amino acids H376 through V483). A [b] indicates that EUCAST susceptibility testing was not performed. A [C] indicates that all isolates of the given species are wild-type. DK-I and DK-II denote novel ITS genotypes identified in this study.

| **Species** | **Terbinafine MIC (mg/L)** | | | | | | | | | | | | | |
|---|---|---|---|---|---|---|---|---|---|---|---|---|---|---|
| SQLE AA alteration | **≤0.004** | **0.004** | **0.008** | **0.016** | **0.03** | **0.06** | **0.125** | **0.25** | **0.5** | **1** | **2** | **4** | **>4** | **Total** |
| ***T. indotineae*** | | | | | **2** | **5** | **2** | | **2** | **9** | **13** | **23** | **49** | **105** |
| WT | | | | | 1 | 2 | | | | | | | | 3 |
| L393F | | | | | | | | | | | | | 1 | 1 |
| L393S | | | | | | | | | 1 | 2 | | | | 3 |
| F397L | | | | | | | | | | | 2 | 17 | 44 | 63 |
| F397L/Y414H | | | | | | | | | | | | | 1 | 1 |
| F397L/A448T | | | | | | | | | | | | 3 | 3 | 6 |
| Q408L | | | | | | | | | | 6 | 11 | 3 | | 20 |
| F415C | | | | | | | | | | 1 | | | | 1 |
| F415L | | | | | | | | | 1 | | | | | 1 |
| A448T | | | | | 1 | 3 | 2 | | | | | | | 6 |
| ***T. interdigitale*** | **2** | **3** | **7** | **7** | **1** | **1** | | **2** | **1** | **1** | | | **1** | **26** |
| WT | 2 | 3 | 7 | 7 | 1 | 1 | | | | | | | | 21 |
| L393F | | | | | | | | | | | | | 1 | 1 |
| L393S | | | | | | | | 1 | 1 | 1 | | | | 3 |
| F415L | | | | | | | | 1 | | | | | | 1 |
| ***T. mentagrophytes* ITS genotype unassigned-I [c] (DK-I)** | | | | | | **1** | | | | | | | | **1** |
| WT (Intrinsic SNPs: N276[a], F419 and T454) | | | | | | 1 | | | | | | | | 1 |
| ***T. mentagrophytes* ITS genotype unassigned-II [c] (DK-II)** | | | **1** | **1** | **1** | **1** | | | | | | | | **4** |
| WT (Intrinsic SNPs: N276[a], F419 and P454) | | | 1 | 1 | 1 | 1 | | | | | | | | 4 |
| ***T. mentagrophytes* ITS genotype III [c]** | | | | **1** | **1** | | | | | | | | | **2** |
| WT (Intrinsic SNPs: N276[a], F419 and T454) | | | | 1 | 1 | | | | | | | | | 2 |
| ***T. mentagrophytes* ITS genotype III* [c]** | | | **1** | **6** | **2** | | | | | | | | | **9** |

| **Species** | **Terbinafine MIC (mg/L)** | | | | | | | | | | | | | |
|---|---|---|---|---|---|---|---|---|---|---|---|---|---|---|
| SQLE AA alteration | **≤0.004** | **0.004** | **0.008** | **0.016** | **0.03** | **0.06** | **0.125** | **0.25** | **0.5** | **1** | **2** | **4** | **>4** | **Total** |
| WT (Intrinsic SNPs:N276[a], F419 and T454) | | | 1 | 6 | 2 | | | | | | | | | 9 |
| ***T. mentagrophytes* ITS genotype IV [c]** | | | | | 1 | | | | | | | | | **1** |
| WT (Intrinsic SNPs:K276[a], L419 and T454) | | | | | 1 | | | | | | | | | 1 |
| ***T. mentagrophytes* ITS genotype VII [c]** | | | | **1** | **1** | | | | | | | | | **2** |
| WT (Intrinsic SNPs:N276[a], F419 and T454) | | | | 1 | 1 | | | | | | | | | 2 |
| ***T. rubrum*** | **7** | **9** | **39** | **61** | **36** | **4** | **7** | **6** | **13** | **15** | **23** | **36** | **29** | **285** |
| WT | 7 | 8 | 38 | 58 | 31 | 1 | | | | | | | | 143 |
| Y110C[a] | | | 1 | | | | | | | | | | | 1 |
| I121M[a] | | | | | 1 | | | | | | | | | 1 |
| I121M[a]/F415S | | | | | | | | | 1 | | | | | 1 |
| L335F[a]/F415S | | | | | | | | | | | 1 | | | 1 |
| L393del | | | | | | | | | | | | 2 | | 2 |
| L393F | | | | | | | | | | | | | 7 | 7 |
| L393F/Y394del | | | | | | | | | | | | | 2 | 2 |
| L393S | | | | | | | | 4 | 5 | 4 | 2 | | | 15 |
| S395_I396insS | | | | | | | | | 3 | | | | | 3 |
| F397I | | | | | | | | 1 | 4 | 6 | | | | 11 |
| F397L | | | | | | | | | | 4 | 20 | 34 | 20 | 78 |
| Y414H | | | | | 1 | 1 | | | | | | | | 2 |
| F415L | | | | | | | 1 | | | | | | | 1 |
| F415S | | | | | | | 1 | 1 | | 1 | | | | 3 |
| H440Y | | | | 2 | 1 | 2 | 4 | | | | | | | 9 |
| A448T | | | | | 1 | | | | | | | | | 1 |
| A448V | | 1 | | | | | | | | | | | | 1 |
| L451P | | | | | | | 1 | | | | | | | 1 |
| C476Y | | | | 1 | 1 | | | | | | | | | 2 |
| ***T. soudanense*[C]** | **1** | | **1** | | | | | | | | | | | **2** |
| ***T. tonsurans*[C]** | | | **6** | **7** | **1** | **2** | | | | | | | | **16** |
| ***T. violaceum*[C]** | | **1** | **3** | **6** | **7** | **4** | **3** | | | | | | | **24** |
| ***T. equinum*[C]** | | | | | **1** | | | | | | | | | **1** |
| ***T. simii*[C]** | **1** | | | | | | | | | | | | | **1** |
| ***T. erinacei*[b,C]** | | | | | | | | | | | | | | **2** |
| ***T. concentricum*[b,C]** | | | | | | | | | | | | | | **1** |
| ***T. europaeum*[C]** | | | | | **1** | | | | | | | | | **1** |
| ***T. benhamiae (var. benhamiae/var. luteum)*[C]** | | **1** | | **6** | **2** | **1** | | | | | | | | **10** |

**Appendix Table 2.** Validation of the full-length *sqle* reference dataset across *Trichophyton* species. Species identity was confirmed by ITS sequencing. Novel full-length *sqle* sequences were generated where unavailable; genome-derived sequences were manually curated and validated against experimental data. *Microsporum canis sqle* served as an outgroup. Novel genotypes were designated DK-I and DK-II. Corresponding ITS sequences (GenBank accession numbers PZ611750 and PZ611751) and sqle sequences (GenBank accession numbers PZ614143–PZ614159 and PX205352) have been deposited in GenBank. The T. schoenleinii sqle sequence was derived from a published genome assembly and was therefore not submitted separately. The number of isolates per species or genotype is indicated; within each group, sqle sequences were identical and correspond to the wild-type allele.

| No. | Species / Genotype | Validation Method | Reference / Comparator Sequences (GenBank) | Notes / Comments |
|---|---|---|---|---|
| **1–4** | ***Trichophyton mentagrophytes*** ITS genotypes III (n = 2), ITS genotype III* (n = 9), ITS genotype IV (n = 1), ITS genotype VII (n = 2) | 100% ITS similarity - Species ID confirmed | MK450325 (ITS) MK447605 (ITS) MK447607 (ITS) MK450323 (ITS) | Public *sqle* sequences excluded due to taxonomic ambiguities and recurrent misclassifications. Novel *sqle* sequences experimentally generated in this study and deposited in GenBank (accession numbers PZ614145–PZ614148). . |
| **5–6** | ***Trichophyton mentagrophytes*** ITS genotype unassigned -I (DK-I), (n = 1) ITS genotype unassigned -II (DK-II), (n = 4) | ~99.4% ITS similarity to known ITS genotypes. | | Distinct ITS consensus not fully matching any reference ITS genotype; included for representation. Novel *sqle* sequences generated in this study and deposited in GenBank (sqle: PZ614143 and PZ614144; ITS: PZ611750 and PZ611751).. |
| **7** | ***Trichophyton indotineae*** (n = 3) | 100% ITS similarity - Species ID confirmed | NR_173767.1 (ITS) | Experimentally generated *sqle* sequence and deposited in GenBank (accession number PZ614149). |
| **8** | ***Trichophyton interdigitale*** (n = 21) | 100% ITS similarity - Species ID confirmed. | Z98001.1 (ITS) KK204440 (*sqle*) | Experimentally generated *sqle* sequence is in complete concordance with curated GenBank *sqle* sequence (accession number PZ614150).. |
| **9** | ***Trichophyton tonsurans*** (n = 16) | 100% ITS similarity - Species ID confirmed | ON791589 (ITS) No publicly available full-length *sqle*. | Novel full-length *sqle* generated in this study and deposited in GenBank (accession number PZ614154).sqle retrieved from annotated genome assembly of strain CBS 112818 (Genbank: GCA_000151455, scaffold GG698517.1; locus tag: TESG_06381) with two exons at positions 103837–104105 and 104168–105368 (reverse strand), separated by a 62 bp intron. Complete concordance between assembly-derived and experimentally sequenced *sqle*. |
| **10** | ***Trichophyton equinum*** (n = 1) | 100% ITS similarity - Species ID confirmed | MH862816 (ITS) No publicly available full-length *sqle*. | Novel full-length *sqle* generated in this study and deposited in GenBank (accession number PZ614155). sqle gene retrieved from annotated genome assembly of strain CBS 127.97 (Genbank: GCA_000151175, scaffold DS995757.1, locus tag: TEQG_06264), with two exons (positions 11417–12617 and 12680–12948 on the forward strand) separated by a 62-bp intron. Complete concordance between assembly-derived and experimentally sequenced *sqle*. |
| **11** | ***Trichophyton schoenleinii*** | Uncultured | No publicly available full-length *sqle*. | *sqle* coding region identified in silico from genome assembly GCA_018357685.2 (scaffold WOFF02000002.1), with two exons at positions 971212–970014 and 969952–969683 on the reverse strand. The full coding sequence was reconstructed from the reverse complement and used as the *sqle* reference for this species. |
| **12** | ***Trichophyton violaceum*** (n = 24) | 100% ITS similarity - Species ID confirmed | AJ270811 (ITS) No publicly available full-length *sqle*. | Novel full-length *SQLE* generated in this study and deposited in GenBank (accession number PZ614153). . *SQLE* coding sequence retrieved from annotated genome assembly (Locus tag: A7D00_2844; GenBank: LHPN01000003), with two exons (positions 1277854–1279054 and 1279117–1279385). Complete concordance between assembly-derived and experimentally sequenced *sqle*. |
| **13** | ***Trichophyton rubrum*** (n = 143) | 100% ITS similarity - | OM313296 (ITS) OM313296 (*sqle*) | Full-length sqle sequence generated in this study and deposited in GenBank (accession number |

| No. | Species / Genotype | Validation Method | Reference / Comparator Sequences (GenBank) | Notes / Comments |
|---|---|---|---|---|
| | | Species ID confirmed | | PZ614151). Complete concordance with curated GenBank *sqle* sequence. |
| **14** | ***Trichophyton soudanense*** (n = 2) | 100% ITS similarity - Species ID confirmed | MK806626 (ITS) No publicly available full-length *sqle*. | Novel full-length *sqle* generated in this study and deposited in GenBank (accession number PZ614152).<br>sqle gene retrieved from genome assembly of strain CBS 452.61(Genbank: GCA_000616865.1, scaffold KK208813.1), two exons at positions 12158–12427 and 12490–13689 on the reverse strand, separated by 62 bp intron. Sequence reconstructed by reverse complementation.<br>Complete concordance between assembly-derived and experimentally sequenced *sqle*. |
| **15** | ***Trichophyton concentricum*** (n = 1) | 100% ITS similarity - Species ID confirmed | LR794126 (ITS) No publicly available full-length *sqle*. | Novel full-length *sqle* generated from isolate CBS109405 in this study and deposited in GenBank (accession number PX205352).<br>Coding sequence manually delineated from the unannotated genome assembly (GenBank: GCA_050494205.1) on scaffold CM115134.1, spanning two exons (4223598–4224797 and 4224861–4225130) separated by a 62 bp intron.<br>Complete concordance between assembly-derived and experimentally sequenced *sqle.* |
| **16** | ***Trichophyton benhamiae sensu stricto*** (n = 10) | 100% ITS similarity - Species ID confirmed | LR794129-LR794131 (ITS) No publicly available full-length *sqle*. | Novel full-length *sqle* generated in this study and deposited in GenBank (accession number PZ614159). |
| **17** | ***Trichophyton europaeum*** (n = 1) | 100% ITS similarity - Species ID confirmed | LR794134, MZ710118, OU231018 (ITS) No publicly available full-length *sqle*. | Novel full-length *sqle* generated in this study and deposited in GenBank (accession number PZ614157).. |
| **18** | ***Trichophyton erinacei*** (n = 2) | 100% ITS similarity - Species ID confirmed | KJ606082, LR794139, MN737936 (ITS) No publicly available full-length *sqle*. | Novel full-length *sqle* generated in this study and deposited in GenBank (accession number PZ614158). |
| **19** | ***Trichophyton simii*** | ITS-confirmed species ID | MH858665 (ITS) No publicly available full-length *sqle*. | Novel full-length *sqle* generated in this study and deposited in GenBank (accession number PZ614156). |
| **20** | Outgroup / ***Microsporum canis*** | - | LC348388 (*sqle*) | Used as outgroup; GenBank *sqle* sequence LC348388. |

**Appendix Table 3**. Pairwise single nucleotide polymorphism (SNP) differences among *Trichophyton* species and genotypes based on full-length *sqle* sequences. Pairwise SNP differences are shown among *Trichophyton* species and genotypes, including defined ITS genotypes and novel lineages (DK-I and DK-II), with *Microsporum canis* as an outgroup.

| **Species complex** | **No.** | **Species / ITS genotype** | **1** | **2** | **3** | **4** | **5** | **6** | **7** | **8** | **9** | **10** | **11** | **12** | **13** | **14** | **15** | **16** | **17** | 18 | **19** | **20** |
|---|---|---|---|---|---|---|---|---|---|---|---|---|---|---|---|---|---|---|---|---|---|---|
| *T. mentagrophytes* complex | 1 | Unassigned lineage-I (DK-I) | | 1 | 0 | 1 | 7 | 0 | 4 | 8 | 11 | 11 | 64 | 64 | 69 | 70 | 70 | 91 | 90 | 91 | 86 | 350 |
| | 2 | Unassigned lineage-II (DK-II) | 1 | | 1 | 2 | 8 | 1 | 5 | 9 | 12 | 12 | 65 | 65 | 70 | 71 | 71 | 92 | 91 | 92 | 87 | 351 |
| | 3 | *ITS genotype* III | 0 | 1 | | 1 | 7 | 0 | 4 | 8 | 11 | 11 | 64 | 64 | 69 | 70 | 70 | 91 | 90 | 91 | 86 | 350 |
| | 4 | ITS genotype III* | 1 | 2 | 1 | | 8 | 1 | 5 | 9 | 12 | 12 | 65 | 65 | 70 | 71 | 71 | 92 | 91 | 92 | 87 | 351 |
| | 5 | ITS genotype IV | 7 | 8 | 7 | 8 | | 7 | 3 | 9 | 8 | 8 | 61 | 61 | 66 | 67 | 67 | 87 | 86 | 87 | 83 | 347 |
| | 6 | *ITS genotype* VII | 0 | 1 | 0 | 1 | 7 | | 4 | 8 | 11 | 11 | 64 | 64 | 69 | 70 | 70 | 91 | 90 | 91 | 86 | 350 |
| | 7 | *T. indotineae* | 4 | 5 | 4 | 5 | 3 | 4 | | 6 | 7 | 7 | 60 | 60 | 65 | 66 | 66 | 88 | 87 | 88 | 83 | 349 |
| | 8 | *T. interdigitale* | 8 | 9 | 8 | 9 | 9 | 8 | 6 | | 13 | 13 | 64 | 66 | 71 | 72 | 72 | 91 | 90 | 91 | 88 | 350 |
| *T. tonsurans* complex | 9 | *T. tonsurans* | 11 | 12 | 11 | 12 | 8 | 11 | 7 | 13 | | 2 | 65 | 65 | 69 | 70 | 70 | 91 | 90 | 91 | 88 | 351 |
| | 10 | *T. equinum* | 11 | 12 | 11 | 12 | 8 | 11 | 7 | 13 | 2 | | 63 | 63 | 69 | 70 | 70 | 91 | 90 | 91 | 88 | 350 |
| | 11 | *T. simii* | 64 | 65 | 64 | 65 | 61 | 64 | 60 | 64 | 65 | 63 | | 24 | 74 | 74 | 74 | 88 | 87 | 88 | 87 | 341 |
| | 12 | *T. schoenleinii* | 64 | 65 | 64 | 65 | 61 | 64 | 60 | 66 | 65 | 63 | 24 | | 75 | 75 | 75 | 90 | 89 | 92 | 90 | 349 |
| *T. rubrum* complex | 13 | *T. violaceum* | 69 | 70 | 69 | 70 | 66 | 69 | 65 | 71 | 69 | 69 | 74 | 75 | | 2 | 2 | 66 | 67 | 71 | 63 | 343 |
| | 14 | *T. rubrum* | 70 | 71 | 70 | 71 | 67 | 70 | 66 | 72 | 70 | 70 | 74 | 75 | 2 | | 0 | 66 | 67 | 71 | 65 | 343 |
| | 15 | *T. soudanense* | 70 | 71 | 70 | 71 | 67 | 70 | 66 | 72 | 70 | 70 | 74 | 75 | 2 | 0 | | 66 | 67 | 71 | 65 | 343 |
| *T. benhamiae* complex | 16 | *T. benhamiae* s.s | 91 | 92 | 91 | 92 | 87 | 91 | 88 | 91 | 91 | 91 | 88 | 90 | 66 | 66 | 66 | | 1 | 10 | 42 | 347 |
| | 17 | *T. concentricum* | 90 | 91 | 90 | 91 | 86 | 90 | 87 | 90 | 90 | 90 | 87 | 89 | 67 | 67 | 67 | 1 | | 9 | 41 | 348 |
| | 18 | *T. europaeum* | 91 | 92 | 91 | 92 | 87 | 91 | 88 | 91 | 91 | 91 | 88 | 92 | 71 | 71 | 71 | 10 | 9 | | 46 | 346 |
| | 19 | *T. erinacei* | 86 | 87 | 86 | 87 | 83 | 86 | 83 | 88 | 88 | 88 | 87 | 90 | 63 | 65 | 65 | 42 | 41 | 46 | | 347 |
| Out-group | 20 | *Microsporum canis* | 350 | 351 | 350 | 351 | 347 | 350 | 349 | 350 | 351 | 350 | 341 | 349 | 343 | 343 | 343 | 347 | 348 | 346 | 347 | |

**Appendix Table 4.** Informative SNP positions used to discriminate *T. indotineae*, *T. interdigitale*, and lineages/genotypes within the *T. mentagrophytes* species complex based on full-length *sqle* sequences. SNP nomenclature is based on the nucleotide present in *T. indotineae*, and positions are derived from a multiple sequence alignment of reference *sqle* sequences. *Bold font indicates the least common nucleotide at a given position, whereas underlined nucleotides indicate positions unique to a single lineage/genotype.

| SNP | *Trichophyton indotineae* | *T. interdigitale* | *T. mentagrophytes* DK-I | *T. mentagrophytes* DK-II | *T. mentagrophytes* III | *T. mentagrophytes* III* | *T. mentagrophytes* IV | *T. mentagrophytes* VII |
|---|---|---|---|---|---|---|---|---|
| T169C | T | **<u>C*</u>** | T | T | T | T | T | T |
| C246T | C | C | C | C | C | C | **<u>T</u>** | C |
| C642T | C | **<u>T</u>** | C | C | C | C | C | C |
| G717A | G | G | G | G | G | **<u>A</u>** | G | G |
| C813T | C | C | C | C | C | C | **<u>T</u>** | C |
| G828C | **G** | **G** | C | C | C | C | **G** | C |
| A882G | A | **<u>G</u>** | A | A | A | A | A | A |
| T996C | **T** | **T** | C | C | C | C | **T** | C |
| T1251G | **T** | G | G | G | G | G | **T** | G |
| C1317T | **C** | **C** | T | T | T | T | **C** | T |
| C1391T | C | C | C | C | C | C | **<u>T</u>** | C |
| A1422C | A | A | A | <u>C</u> | A | A | A | A |
| T1457G | T | **<u>G</u>** | T | T | T | T | T | T |
| C1460T | C | **<u>T</u>** | C | C | C | C | C | C |

**Appendix Table 5.** Pairwise single nucleotide polymorphism (SNP) differences among *Trichophyton* species and genotypes based on the partial 388-bp *sqle* fragment. Pairwise SNP differences are shown among *Trichophyton* species and genotypes, including defined ITS genotypes and novel lineages (DK-I and DK-II), with Microsporum canis included as an outgroup. The analyzed fragment (bp 1125–1512) encompasses the clinically relevant terbinafine resistance hotspot region.

| **Species complex** | **No.** | **Species / ITS genotype** | **1** | **2** | **3** | **4** | **5** | **6** | **7** | **8** | **9** | **10** | **11** | **12** | **13** | **14** | **15** | **16** | **17** | 18 | **19** | **20** |
|---|---|---|---|---|---|---|---|---|---|---|---|---|---|---|---|---|---|---|---|---|---|---|
| *Trichophyton mentagrophytes* complex | 1 | Genotype unassigned-I (DK-I) | | 1 | 0 | 0 | 3 | 0 | 2 | 3 | 4 | 3 | 20 | 22 | 22 | 22 | 22 | 25 | 25 | 25 | 25 | 121 |
| | 2 | Genotype unassigned-II (DK-II) | 1 | | 1 | 1 | 4 | 1 | 3 | 4 | 5 | 4 | 21 | 23 | 23 | 23 | 23 | 26 | 26 | 26 | 26 | 122 |
| | 3 | *ITS genotype* III | 0 | 1 | | 0 | 3 | 0 | 2 | 3 | 4 | 3 | 20 | 22 | 22 | 22 | 22 | 25 | 25 | 25 | 25 | 121 |
| | 4 | ITS genotype III* | 0 | 1 | 0 | | 3 | 0 | 2 | 3 | 4 | 3 | 20 | 22 | 22 | 22 | 22 | 25 | 25 | 25 | 25 | 121 |
| | 5 | ITS genotype IV | 3 | 4 | 3 | 3 | | 3 | 1 | 4 | 3 | 2 | 19 | 21 | 21 | 21 | 21 | 23 | 23 | 23 | 24 | 119 |
| | 6 | *ITS genotype* VII | 0 | 1 | 0 | 0 | 3 | | 2 | 3 | 4 | 3 | 20 | 22 | 22 | 22 | 22 | 25 | 25 | 25 | 25 | 121 |
| | 7 | *T. indotineae* | 2 | 3 | 2 | 2 | 1 | 2 | | 3 | 2 | 1 | 18 | 20 | 20 | 20 | 20 | 24 | 24 | 24 | 24 | 120 |
| | 8 | *T. interdigitale* | 3 | 4 | 3 | 3 | 4 | 3 | 3 | | 5 | 4 | 21 | 23 | 23 | 23 | 23 | 26 | 26 | 26 | 26 | 121 |
| *Trichophyton tonsurans* complex | 9 | *T. tonsurans* | 4 | 5 | 4 | 4 | 3 | 4 | 2 | 5 | | 1 | 20 | 22 | 21 | 21 | 21 | 26 | 26 | 26 | 26 | 121 |
| | 10 | *T. equinum* | 3 | 4 | 3 | 3 | 2 | 3 | 1 | 4 | 1 | | 19 | 21 | 20 | 20 | 20 | 25 | 25 | 25 | 25 | 120 |
| | 11 | *T. simii* | 20 | 21 | 20 | 20 | 19 | 20 | 18 | 21 | 20 | 19 | | 6 | 21 | 21 | 21 | 27 | 27 | 27 | 27 | 120 |
| | 12 | *T. schoenleinii* | 22 | 23 | 22 | 22 | 21 | 22 | 20 | 23 | 22 | 21 | 6 | | 23 | 23 | 23 | 29 | 29 | 29 | 29 | 122 |
| *Trichophyton rubrum* complex | 13 | *T. violaceum* | 22 | 23 | 22 | 22 | 21 | 22 | 20 | 23 | 21 | 20 | 21 | 23 | | 0 | 0 | 20 | 20 | 20 | 21 | 118 |
| | 14 | *T. rubrum* | 22 | 23 | 22 | 22 | 21 | 22 | 20 | 23 | 21 | 20 | 21 | 23 | 0 | | 0 | 20 | 20 | 20 | 21 | 118 |
| | 15 | *T. soudanense* | 22 | 23 | 22 | 22 | 21 | 22 | 20 | 23 | 21 | 20 | 21 | 23 | 0 | 0 | | 20 | 20 | 20 | 21 | 118 |
| *Trichophyton benhamiae* complex | 16 | *T. benhamiae* s.s | 25 | 26 | 25 | 25 | 23 | 25 | 24 | 26 | 26 | 25 | 27 | 29 | 20 | 20 | 20 | | 0 | 0 | 14 | 119 |
| | 17 | *T. concentricum* | 25 | 26 | 25 | 25 | 23 | 25 | 24 | 26 | 26 | 25 | 27 | 29 | 20 | 20 | 20 | 0 | | 0 | 14 | 119 |
| | 18 | *T. europaeum* | 25 | 26 | 25 | 25 | 23 | 25 | 24 | 26 | 26 | 25 | 27 | 29 | 20 | 20 | 20 | 0 | 0 | | 14 | 119 |
| | 19 | *T. erinacei* | 25 | 26 | 25 | 25 | 24 | 25 | 24 | 26 | 26 | 25 | 27 | 29 | 21 | 21 | 21 | 14 | 14 | 14 | | 122 |
| Out-group | 20 | *Microsporum canis* | 121 | 122 | 121 | 121 | 119 | 121 | 120 | 121 | 121 | 120 | 120 | 122 | 118 | 118 | 118 | 119 | 119 | 119 | 122 | |

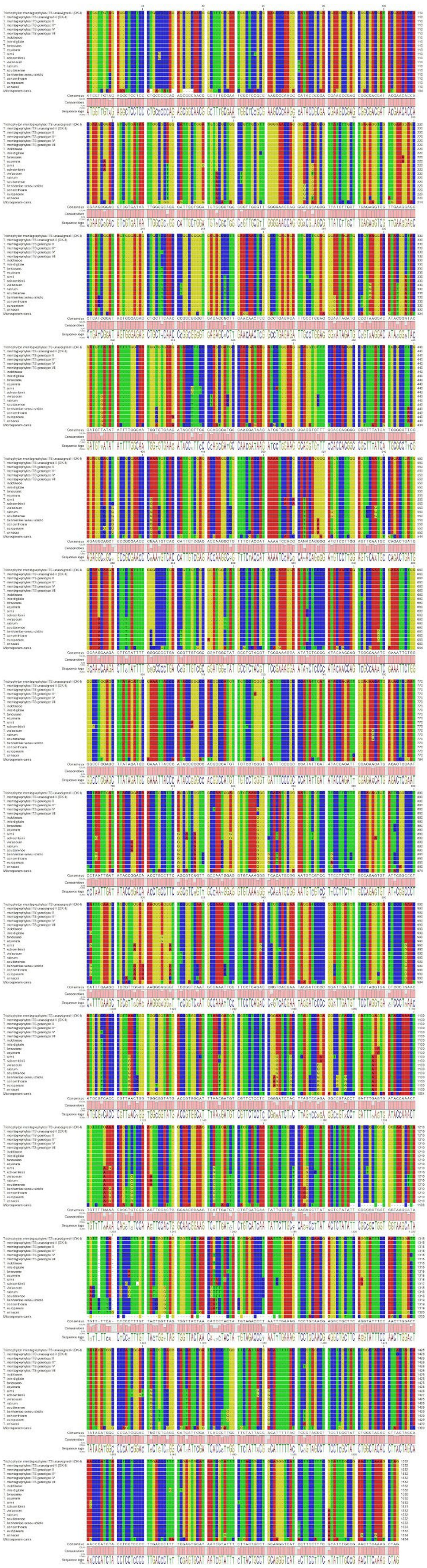

**Appendix Figure 1.** Multiple sequence alignment of the full-length ***sqle*** gene across ***Trichophyton*** species. Nucleotide alignment showing interspecies variation across the gene. Identical residues are indicated by dots; variable positions are shown as nucleotides. Single nucleotide polymorphisms are well defined at specific positions.

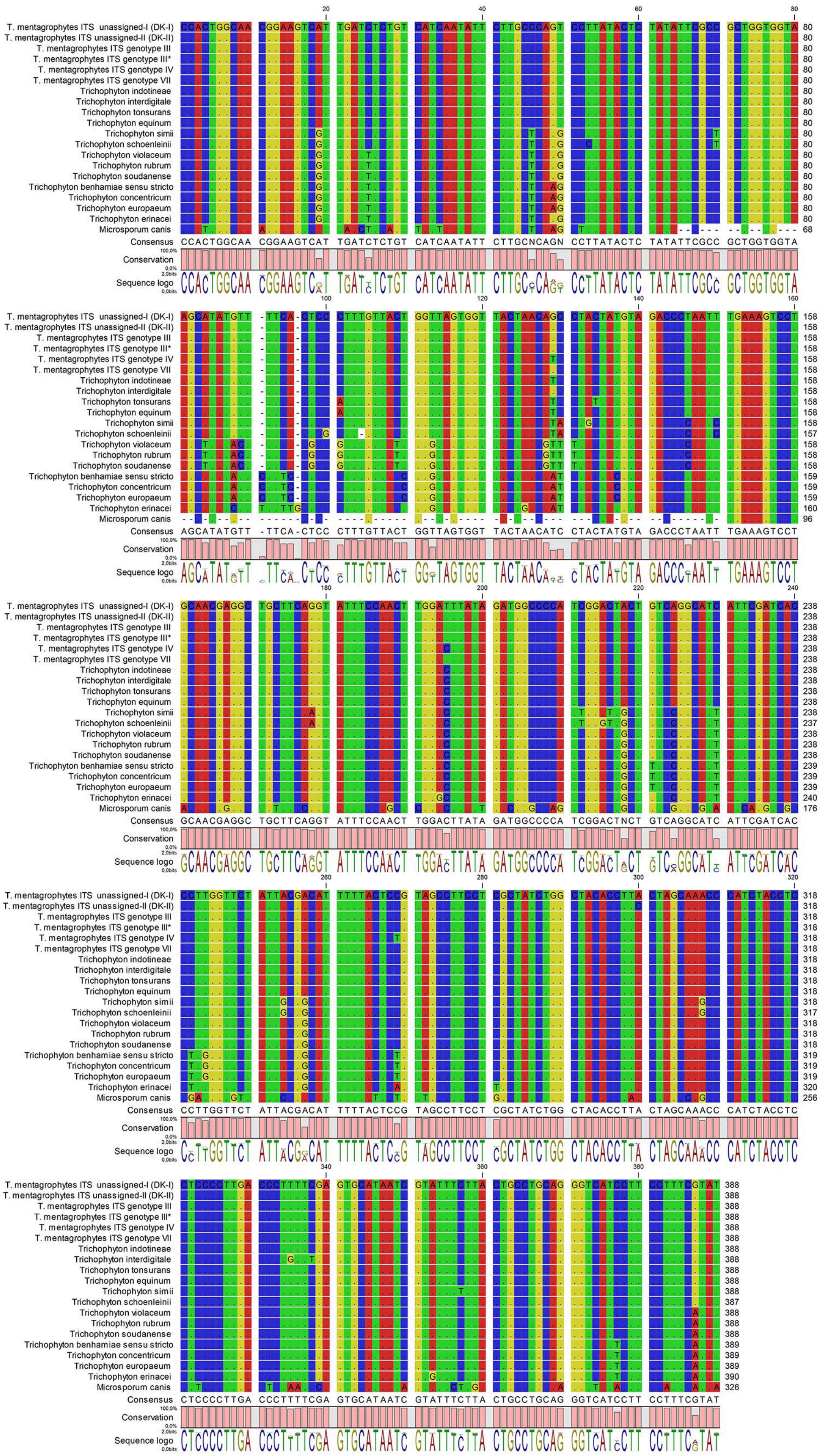


**Appendix Figure 2.** Multiple sequence alignment of a 388-bp fragment used for partial *sqle* PCR across *Trichophyton* species, encompassing terbinafine resistance-associated hotspots. Identical residues are indicated by dots; variable positions are shown as nucleotides. Species-discriminating SNPs occur at specific positions within the fragment.
